# Galectin-3 Knockdown Impairs Survival, Migration, and Immunomodulatory Actions of Mesenchymal Stromal Cells in a Mouse Model of Chagas Disease Cardiomyopathy

**DOI:** 10.1155/2017/3282656

**Published:** 2017-07-10

**Authors:** Bruno Solano de Freitas Souza, Kátia Nunes da Silva, Daniela Nascimento Silva, Vinícius Pinto Costa Rocha, Bruno Diaz Paredes, Carine Machado Azevedo, Carolina Kymie Nonaka, Gisele Batista Carvalho, Juliana Fraga Vasconcelos, Ricardo Ribeiro dos Santos, Milena Botelho Pereira Soares

**Affiliations:** ^1^Gonçalo Moniz Institute, FIOCRUZ, Salvador, BA, Brazil; ^2^Center for Biotechnology and Cell Therapy, São Rafael Hospital, Salvador, BA, Brazil

## Abstract

Therapies based on transplantation of mesenchymal stromal cells (MSC) hold promise for the management of inflammatory disorders. In chronic Chagas disease cardiomyopathy (CCC), caused by chronic infection with *Trypanosoma cruzi*, the exacerbated immune response plays a critical pathophysiological role and can be modulated by MSC. Here, we investigated the role of galectin-3 (Gal-3), a beta-galactoside-binding lectin with several actions on immune responses and repair process, on the immunomodulatory potential of MSC. Gal-3 knockdown in MSC did not affect the immunophenotype or differentiation potential. However, Gal-3 knockdown MSC showed decreased proliferation, survival, and migration. Additionally, when injected intraperitoneally into mice with CCC, Gal-3 knockdown MSC showed impaired migration in vivo. Transplantation of control MSC into mice with CCC caused a suppression of cardiac inflammation and fibrosis, reducing expression levels of CD45, TNF*α*, IL-1*β*, IL-6, IFN*γ*, and type I collagen. In contrast, Gal-3 knockdown MSC were unable to suppress the immune response or collagen synthesis in the hearts of mice with CCC. Finally, infection with *T. cruzi* demonstrated parasite survival in wild-type but not in Gal-3 knockdown MSC. These findings demonstrate that Gal-3 plays a critical role in MSC survival, proliferation, migration, and therapeutic potential in CCC.

## 1. Introduction

Mesenchymal stromal cells (MSC) are multipotent stem cells with the ability to differentiate into mesoderm-derived cell lineages, such as chondrocytes, osteocytes, and adipocytes [1]. Described by Friedenstein and colleagues in 1970 [2], MSC are plastic-adherent cells presenting fibroblast-like morphology and are characterized by the expression of specific surface markers and demonstration of trilineage differentiation potential. MSC can be easily obtained from different organs and tissues of adult individuals, being presently among the most studied cell types in cell therapies [1].

The potential use of MSC to treat inflammatory and autoimmune disorders is based on several described immunomodulatory actions, including inhibition of the activation of T and B lymphocytes, NK cells, and dendritic cells and stimulation of regulatory T cell differentiation [3]. The anti-inflammatory actions of MSC are well studied and found to be mediated by IL-10, TGF-*β*, PGE2, HGF, and IDO (for human cells) or iNOS (for mouse cells). Galectin-3 (Gal-3) has also been suggested as a critical mediator of immunomodulatory actions of human MSC [4, 5].

Galectins are a group of galactoside-binding lectins that regulate various biological processes. Gal-3 is present in the extracellular and intracellular compartments, being involved in cell adhesion, migration, apoptosis, inflammation, and tissue repair [6]. Expression of Gal-3 in fibroblasts is associated with proliferation and synthesis of extracellular matrix components, contributing to scar formation [7–9]. In endothelial precursor cells, Gal-3 promotes proliferation and angiogenesis [10]. While the role of Gal-3 in immune cells has been extensively studied, the actions affected by Gal-3 expression in MSC are not well established. Being highly expressed in inflammatory and fibrogenic microenvironments in tissues [11], Gal-3 is likely to affect MSC biology and response, naturally or in a cell therapy scenario.

Cell therapy has been investigated as a potential alternative treatment for Chagas disease cardiomyopathy, a relevant cause of chronic heart failure in Latin America which results from *Trypanosoma cruzi* infection [12, 13]. An exacerbated immune response directed against the parasite and to host antigens plays a central role in the pathogenesis of CCC, leading to progressive cardiomyocyte loss, fibrosis, arrhythmia, and loss of ventricular function [13]. Previously, it was demonstrated that transplantation of MSC into mice chronically infected with *T. cruzi* caused a reduction of myocarditis and modulation of fibrosis [14–16]. Additionally, we have shown that Gal-3 expression is increased in the hearts of chronic chagasic mice and in human samples [17, 18]. *T. cruzi* infection induces increased Gal-3 expression in different cell types, which favors parasite adhesion, migration, invasion, and reduces antiparasitic immune responses [19–23]. Here, we investigated the potential involvement of Gal-3 in the ability of MSC to migrate and exert immunomodulatory actions in a mouse model of CCC, also investigating potential actions in parasite-host cell interactions.

## 2. Materials and Methods

### 2.1. Animal Procedures

Six- to eight-week-old female C57BL/6 mice were used in this study. All animals were raised and maintained at the animal facility of the Center for Biotechnology and Cell Therapy, São Rafael Hospital, in rooms with controlled temperature (22 ± 2°C) and humidity (55 ± 10%), continuous air flow, and 12 h light/12 h dark cycles (6 am–6 pm) and provided with rodent diet and water ad libitum. Mice were handled according to the NIH guidelines for animal experimentation, and the study received prior approval by the animal ethics committee at São Rafael Hospital.

### 2.2. Isolation and Culture of MSC

Bone marrow cells were obtained from the tibiae and femurs by flushing and were cultured in Dulbecco's modified Eagle's medium (DMEM; ThermoFisher Scientific, Waltham, MA, USA), 10% fetal bovine serum (ThermoFisher Scientific), and 1% penicillin/streptomycin (ThermoFisher Scientific) in a humidified incubator at 37°C with 5% atmospheric CO_2_. The medium was changed every 2-3 days and, when the culture reached 90% confluency, the cells were passaged with trypsin-EDTA 0.25% solution (ThermoFisher Scientific).

### 2.3. Galectin-3 Knockdown

Stable Gal-3 knockdown was achieved by MSC or J774 macrophages by transduction with lentiviral vectors carrying a shRNA sequence targeting *Lgals3* gene or scrambled control (Lgals3_shRNA1 5′-GATTTCAGGAGAGGGAATGAT-3′; one Lgals3_scrbl_shRNA 5′-AGGTATGAGTCGAGATTGAGA-3′), as previously described [18]. Culture medium was replaced and the cells were cultured for an additional 48 h, being assessed for GFP reporter gene expression by using an inverted fluorescence microscope (Eclipse Ti-E; Nikon, Tokyo, Japan). The cells were expanded and knockdown efficiency for each shRNA was evaluated by confocal microscopy and qPCR analyses.

### 2.4. Flow Cytometry Analysis

For immunophenotyping, MSC lines were passaged and centrifuged and the pellet was resuspended in PBS. A total of 5 × 10^5^ cells was used for labeling with the following antibodies in the concentration 1/50: Sca1PE-Cy7 (BD Biosciences, San Jose, CA, USA), CD45-PerCP (eBioscience, San Diego, CA, USA), CD44-PE (BD Bioscience), CD90-APC (BD Bioscience), CD34-AlexaFluor647 (BD Bioscience), and control isotypes. Cells were incubated in 100 *μ*L of binding buffer (ThermoFisher Scientific) with annexin-V-FITC and 7-AAD (BD Biosciences, San Jose, CA, USA) for 15 minutes in the dark at RT. After the incubation period, cells were washed twice with PBS, and the data acquisition and analysis were performed using a LRSFortessa flow cytometer (BD Biosciences). At least 10,000 events were acquired and analyzed.

### 2.5. Trilineage Differentiation Assay

Adipogenic, osteogenic, and chondrogenic differentiations were performed using commercially available kits, following the manufacturer's instructions (ThermoFisher Scientific). For adipogenic differentiation, cells were cultured in 24-well plates in an adipogenic induction medium, StemPro Adipogenesis Differentiation Kit. Lipid inclusions were detected on differentiation day 14, by fixation in 4% paraformaldehyde and staining with Oil red solution. For osteogenic differentiation, the cells were cultured in a specific osteogenic differentiation medium, StemPro Osteogenesis Differentiation Kit. Half the differentiation medium was changed every two days. Calcium-rich matrix deposition was observed by staining with Alizarin red 2%. For chondrogenic differentiation, cells were cultured for 21 days in chondrogenic differentiation medium, StemPro Chondrogenesis Differentiation Kit. Proteoglycan synthesis was evaluated after staining with Alcian Blue solution. The images were captured with an inverted microscope (Eclipse Ti, Nikon, Tokyo, Japan).

### 2.6. Endothelial Cell Differentiation

Differentiation of MSC to endothelial cells was performed by incubating the cells with EGM-2 medium (Lonza, Basel, Switzerland), as previously described [15]. Endothelial tube formation assay was performed to observe capillary-like 3-D structures by plating the differentiated cells on Matrigel (Corning, Corning, NY, USA). The images were captured using an inverted microscope (Eclipse Ti, Nikon).

### 2.7. Proliferation Assay

For comparative evaluation of the proliferation rate among different MSC lines, the cells were plated in 96-well plates, at a density of 10^4^ cells/well, in a final volume of 200 *μ*L, in triplicate, and cultured in DMEM supplemented with 10% FBS. After 24 h, plates were pulsed with 1 *μ*Ci of methyl-^3^H thymidine (PerkinElmer) for 18 h, and proliferation was assessed by measurement of ^3^H-thymidine uptake by using a Chameleon *β*-plate counter (Hydex; Turku, Finland).

### 2.8. Cell Migration Analyses

MSC were plated in wells of a 24-well plate, at a cell density of 5 × 10^4^ cells/cm^2^. Live cell imaging was performed using the Operetta High Content Imaging System (Perkin Elmer) under controlled temperature (37°C) and atmospheric CO_2_ (5%). Digital phase-contrast images were acquired at 10x magnification (10x high NA objective) using Operetta's automatic digital phase-contrast algorithm. Image acquisition interval was set to 10 min during 16 h. Images were segmented using the Find Cells building block of the Harmony 3.5.2 software (Perkin Elmer), which provides a dedicated algorithm for segmenting digital phase-contrast images. The segmented cells were subjected to cell tracking using the Track Objects building block. Properties that describe cell migration per time point were calculated, such as displacement. Representative graphs of mean square displacement for each well is shown. For in vitro wound healing assay, MSC were cultured in a 6-well plate until a monolayer was formed. A pipette tip was used to make a scratch along the well, and the area was photographed at time point 0 and after 3 days for gap distance measurements.

### *2.9.T. cruzi* Infection and Cell Transplantation

Trypomastigotes of the myotropic Colombian *T. cruzi* strain were obtained from culture supernatants of infected LLC-MK2 cells, as previously described [24]. Then, C57BL/6 mice were infected by intraperitoneal injection with 1000 *T. cruzi* trypomastigotes in PBS. Infection was confirmed by following parasitemia at different time points after infection.

Six months after infection, mice were randomly assigned into three groups: control MSC, Gal-3 knockdown MSC, or saline. The administration regimen consisted of one weekly intraperitoneal injection of a suspension of 10^6^ MSC, or equal volume of saline (100 *μ*L). Mice were euthanized by cervical dislocation under anesthesia with ketamine (100 mg/kg) and xylazine (10 mg/kg), on the 7th week after the beginning of the treatment, for analysis.

For in vitro infections, MSC or J774 macrophages were incubated with *T. cruzi* trypomastigotes (MOI = 10) for 24 h. Then, the wells were washed and the medium replaced. Cells were fixed, stained with DAPI for parasite quantification in the Operetta system (PerkinElmer), or submitted for transmission electron microscopy processing and analysis. For ultrastructural analysis, cells were fixed at 4°C for 12 h in a solution of 3% glutaraldehyde (Sigma-Aldrich) in PBS, washed with 0.1 M sodium cacodylate buffer, and postfixed in osmium tetroxide 1% for 30 min. Dehydration was performed by using a graded series of acetone solutions, then the samples were embedded in epoxy resin Polybed812 (Electron Microscopy Sciences, Hatfield, PA, USA). Ultrathin sections were obtained using EM UC7 ultramicrotome (Leica, Wetzlar, Germany) and contrasted with uranyl acetate and lead citrate. The sections were analyzed using a transmission electron microscope JEM1230 JEOL (Tokyo, Japan) at 80 kV.

### 2.10. Real-Time Reverse Transcription Polymerase Chain Reaction (RT-qPCR)

Dissociated cells, heart, and spleen samples were subjected to total RNA extraction using TRIzol reagent (Thermo Scientific). The RNA concentration was determined by spectrophotometry. Next, cDNA was synthetized, starting with 1 *μ*g RNA using High Capacity cDNA Reverse Transcription Kit (Thermo Scientific), following the manufacturer's instructions. RT-qPCR assays were performed to detect the expression levels of *Tbet* (Mm_00450960_m1), *Tnf* (Mm_00443258_m1), *Ifng* (Mm_00801778_m1), *Col1a1* (Mm_0801666_g1), *Il1b* (Mm_0043228_m1), *Il6* (Mm_00446190_m1), and *Ptprc* (Mm_01293577_m1). The RT-qPCR amplification mixtures contained 20 *η*g template cDNA, Taqman Master Mix (10 *μ*L), and probes in a final volume of 20 *μ*L (all from Thermo Scientific). The reactions were run in duplicate on an ABI7500 Sequence Detection System (Thermo Scientific) under standard thermal cycling conditions. The mean Ct (cycle threshold) values from duplicate measurements were used to calculate the expression of the target gene, with normalization to an internal control—*Gapdh (*mm99999915_g1), using the 2−DCt formula. Experiments with coefficients of variation greater than 5% were excluded. A nontemplate control and nonreverse transcription controls were also included.

### 2.11. Histology and Morphometric Analyses

Hearts were collected and fixed in 10% buffered formalin. Heart sections were analyzed by light microscopy after paraffin embedding, followed by standard hematoxylin and eosin (H&E), or Sirius red staining. Sirius red-stained sections were entirely digitalized using a confocal microscope A1+ (Nikon). The percentage of fibrosis was determined by analysis of whole sections stained with Sirius red-stained heart sections and semiautomatic morphometric quantification using Image Pro Plus v.7.0. Two blinded investigators performed the analyses.

### 2.12. Immunofluorescence Analysis

Immunostainings for detection of Gal-3 expression were performed in MSC plated on coverslips. The cells were fixed with paraformaldehyde 4% and incubated overnight at 4°C with the primary antibody goat anti-Gal-3, diluted 1 : 400 (Santa Cruz Biotechnology, Dallas, TX, USA). On the following day, sections were incubated for 1 h with phalloidin conjugated with Alexa Fluor 488 (1 : 200; ThermoFisher Scientific) mixed with the secondary antibody anti-goat IgG Alexa Fluor 568 (1 : 1000; ThermoFisher Scientific). Proliferating cells were evaluated by KI67 staining (anti-Ki67 1 : 1000; ThermoFisher Scientific), followed by anti-rabbit IgG Alexa Fluor 568 (1 : 1000 ThermoFisher Scientific). Dead cells were stained with PI (BD Biosciences). Nuclei were stained with 4,6-diamidino-2-phenylindole (VECTASHIELD mounting medium with DAPI H-1200; Vector Laboratories, Cambridgeshire, UK). The presence of fluorescent cells was determined by observation using an A1+ confocal microscope (Nikon).

### 2.13. Statistical Analyses

Continuous variables are presented as means ± SEM. Parametric data were analyzed using Student's unpaired *t*-test, for comparisons between two groups, and 1-way ANOVA, followed by Bonferroni post hoc test for multiple-comparison test, using Prism 6.0 (GraphPad Software). Values of *P* < 0.05 were considered statistically significant.

## 3. Results

**Figure 1:**
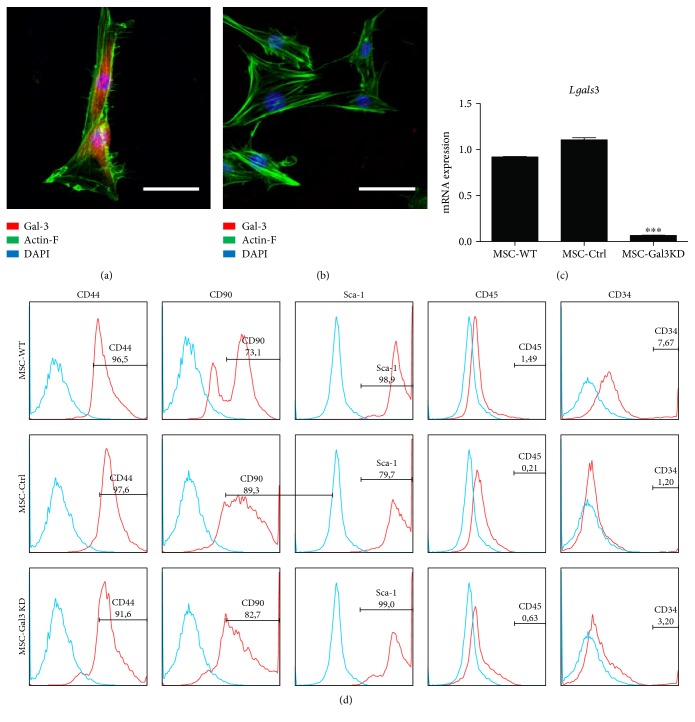
Evaluation of Gal-3 knockdown efficiency and characterization of MSC lines. Confocal microscopy images showing Actin-F (green), Gal-3 (red), and nuclei stained with DAPI (blue) in wild-type (a) and Gal-3 knockdown MSC (b). Scale bars = 20 *μ*m. (c) Gene expression analysis of *Lgals3* by qRT-PCR. ^∗∗∗^*P* < 0.001, compared to the other groups. (d) Histograms demonstrating expression of surface marker characteristics of MSC and low expression of hematopoietic markers, analyzed by flow cytometry.

Bone marrow-derived MSC lines were generated by transduction with lentiviral vectors containing the shRNA sequence targeting Gal-3 gene or a nontargeting scrambled sequence. The MSC lines were assessed for Gal-3 expression, in order to confirm the knockdown efficiency by confocal microscopy and qPCR analysis (Figures 1(a), 1(b), and 1(c)). Gal-3 was expressed in the cytoplasm and inside the nuclei of wild-type (Figure 1(a)) and control vector-transduced MSC lines. Cells transduced with the vector containing the shRNA sequence for Gal-3 knockdown showed a marked reduction of Gal-3 expression (Figure 1(b)). This finding was confirmed quantitatively at the mRNA level by RT-qPCR analysis (Figure 1(c)).

**Figure 2:**
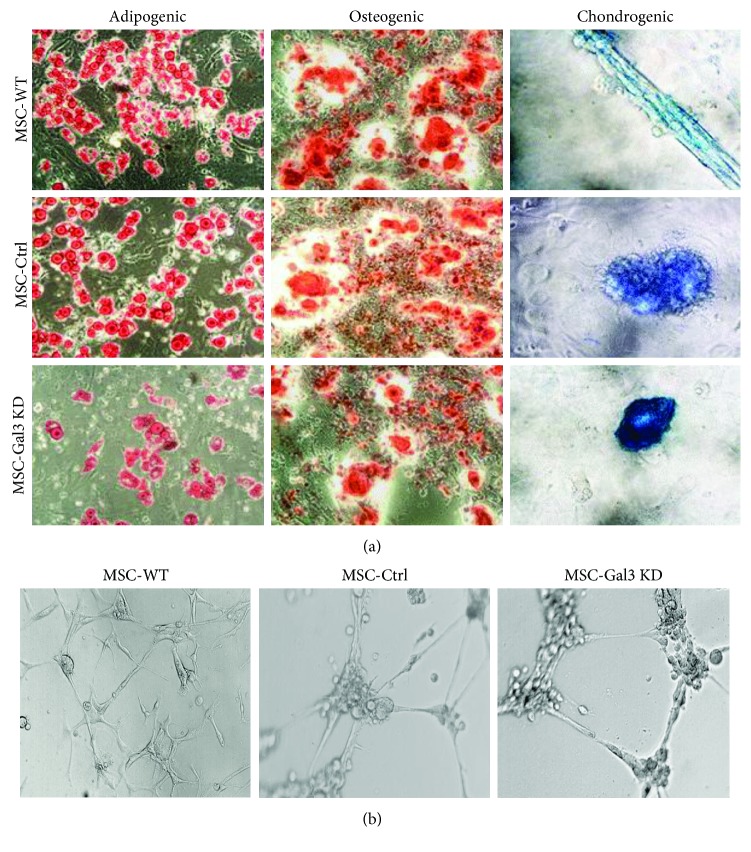
Characterization of MSC lines by differentiation assays. (a) Trilineage differentiation assay performed in MSC lines to generate adipocytes, visualized by Oil red staining, osteocytes, visualized by alizarin red staining, and chondrocytes, visualized by Alcian blue staining, respectively. (b) Angiogenic ability demonstrated by endothelial tube formation assay on Matrigel. MSC-WT = wild-type MSC; MSC-Ctrl = MSC transduced with a nontargeting shRNA vector; MSC-Gal3KD = Gal-3 knockdown MSC. Magnification = 200x.

MSC lines were then characterized in order to ensure the maintenance of the phenotype and biological properties that define MSC. Immunophenotyping by flow cytometry showed a similar pattern of expression of surface markers by the different cell lines, with a positive staining for the MSC markers CD44, CD90, and Sca-1, and low frequency of cells expressing hematopoietic lineage markers CD45 and CD34 (Figure 1(d)). Next, we assessed the multipotential of MSC by a trilineage differentiation assay in vitro. Upon induction by specific culture media, Gal-3 knockdown and control MSC lines were able to efficiently undergo osteogenic, chondrogenic, and adipogenic differentiation (Figure 2(a)). Additionally, knockdown of Gal-3 in MSC did not interfere with their ability to form capillary-like structures when cultured in endothelium-inducer medium (Figure 2(b)).

**Figure 3:**
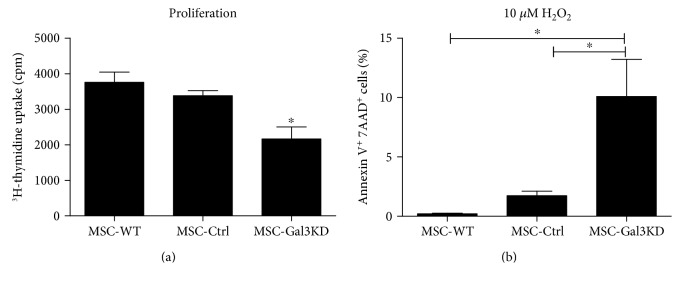
Effects of Gal-3 knockdown on cell proliferation and survival. (a) Proliferation rate in different MSC lines, evaluated by ^3^H-thymidine incorporation assay. ^∗^*P* < 0.05, compared to the other groups. (b) Apoptosis analysis by Annexin V/7-AAD assay, comparing the rate of cells undergoing apoptosis after incubation with 10 *μ*M H_2_O_2_. MSC-WT = wild-type MSC; MSC-Ctrl = MSC transduced with a nontargeting shRNA vector; MSC-Gal3KD = Gal-3 knockdown MSC. ^∗^*P* < 0.05.

Next, MSCs were analyzed regarding proliferation rate and survival. We found that Gal-3 knockdown MSC present decreased proliferation rate when compared to controls, as measured by ^3^H-thymidine incorporation (Figure 3(a)). Moreover, the number of cells undergoing apoptosis was higher in Gal-3 knockdown MSC, when compared to controls, after incubation with 10 μM H_2_O_2_ (Figure 3(b)).

**Figure 4:**
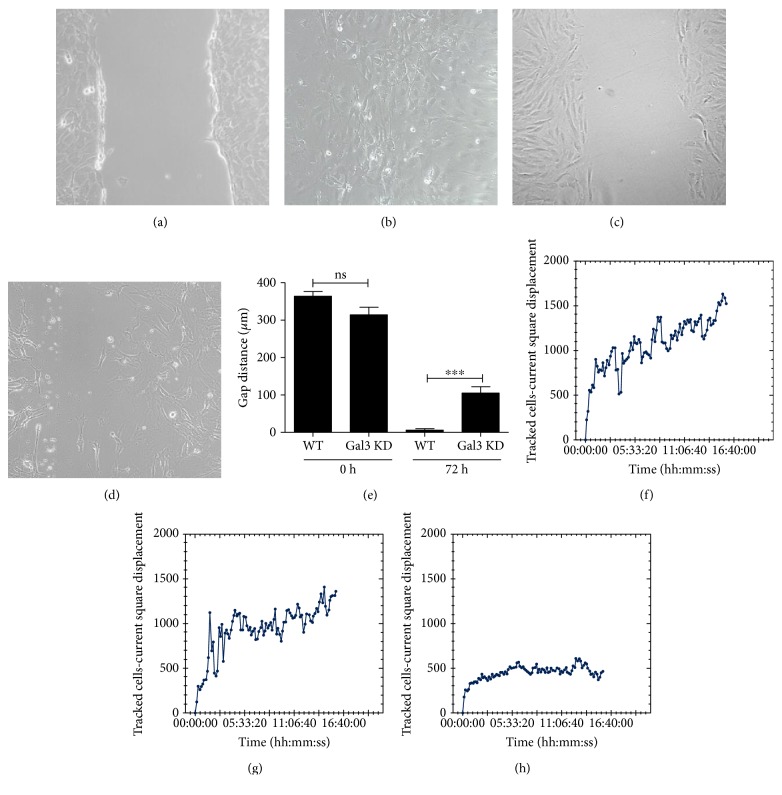
Gal-3 knockdown MSC exhibit defective migration and displacement in vitro. Migration was evaluated by the wound healing assay. Phase contrast representative images showing scratch area at day 0 for wild-type MSC (a) and Gal-3 knockdown MSC (c), and at day 3 for wild-type MSC (b) and Gal-3 knockdown MSC (d). (e) Gap distance was evaluated at 72 h and compared to time 0. ^∗∗∗^*P* < 0.001. (f–h) Mean square displacements were obtained by individually tracked cells at various time points, from the time of the first position, until the end of the overnight incubation of MSC-WT (d), MSC-Ctrl (e), and MSC-Gal3KD (f). MSC-WT = wild-type MSC; MSC-Ctrl = MSC transduced with a nontargeting shRNA vector; MSC-Gal3KD = Gal-3 knockdown MSC.

Galectin 3 is known to affect cell-extracellular matrix protein binding and cell migration processes [25, 26]. To investigate whether Gal-3 knockdown interferes with migration of MSC, we assessed the in vitro migratory ability of Gal-3 knockdown MSC and control cell lines in vitro. Gal-3 knockdown caused a decreased migration in vitro in a wound healing assay when compared to control MSC (Figures 4(a), 4(b), 4(c), 4(d), and 4(e)). Additionally, by using displacement cell tracking by time-lapse image analysis, we found that Gal-3 knockdown caused a reduction in the mobility of MSC when compared to controls (Figures 4(f), 4(g), and 4(h)).

**Figure 5:**
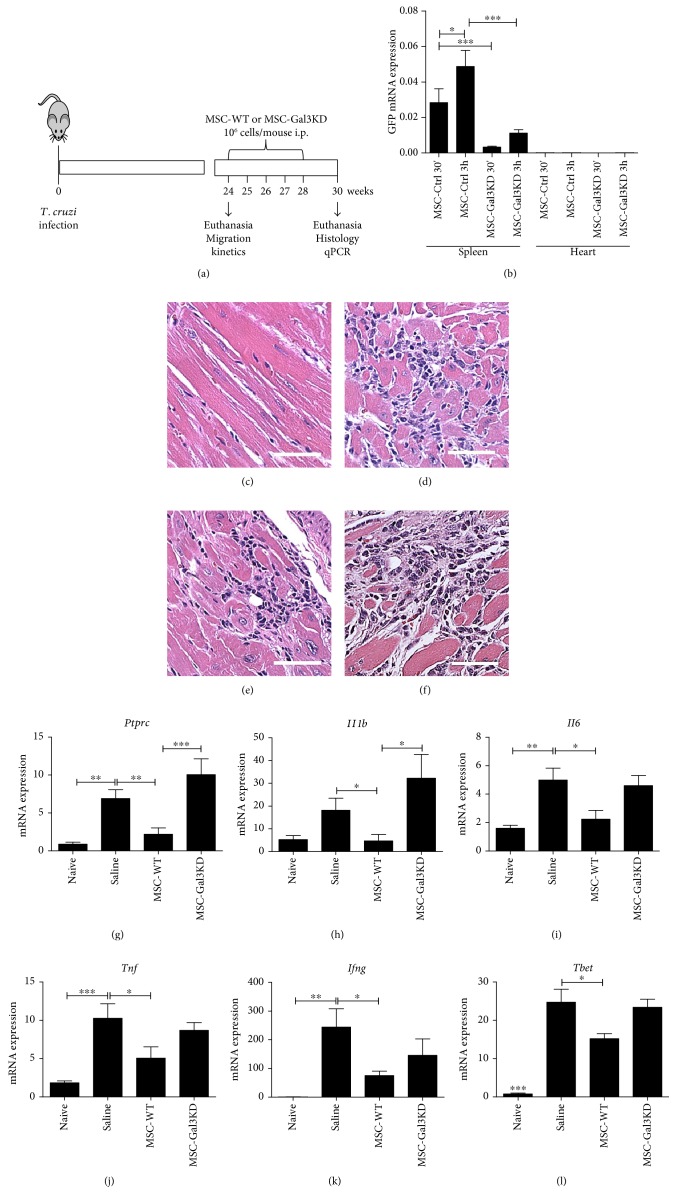
Effects of the transplantation of MSC lines in a mouse model of chronic *T. cruzi* infection. (a) Study design. (b) Cell migration and homing to spleens and hearts were evaluated by amplification of GFP mRNA by qRT-PCR. (c–f) Representative images of H&E stained heart sections of naïve mice (c), infected and administered with saline (d), MSC-WT (e), or MSC-Gal3KD (f). Quantification of mRNA expression levels of CD45 coding gene (PTPRC), evaluated qRT-PCR (g). RTqPCR analysis of gene expression in the heart tissue of the cytokines IL1-*β* (h), IL-6 (i), TNF-*α* (j), IFN-*γ* (k), and Th1-associated transcription factor T-bet (l). ^∗^*P* < 0.05; ^∗∗^*P* < 0.01; ^∗∗∗^*P* < 0.001. MSC-WT = wild-type MSC; MSC-Ctrl = MSC transduced with a nontargeting shRNA vector; MSC-Gal3KD = Gal-3 knockdown MSC.

In order to test if Gal-3 knockdown could impair MSC therapeutic actions in an in vivo setting, the cells were administered i.p. into mice chronically infected with *T. cruzi*, a model of chronic Chagas disease cardiomyopathy (Figure 5(a)). First, the ability of MSC to migrate to the spleen and heart was evaluated shortly after transplantation, by qPCR analysis of GFP mRNA expression. GFP expression was detected in the spleens as early as 30 min after cell transplantation and increased at the 3 h time point. However, significantly lower levels of GFP mRNA expression were detected in the spleens of mice transplanted with Gal-3 knockdown MSC when compared to control MSC, both 30 min and 3 h after the cell administration. Negligible levels of GFP were detected in the hearts at the same time points (Figure 5(b)).

Next, we investigated the long-term effects of cell transplantation in *T. cruzi*-infected mice. Groups of mice received weekly i.p. injections of 10^6^ MSC—wild-type or Gal-3 knockdown cell line—for five weeks. A vehicle control group was injected with equal volumes of saline solution (Figure 5(a)). Seven weeks after the beginning of the treatment, mice were euthanized for histological and molecular evaluations.

Histological analysis of heart sections revealed the presence of multifocal inflammatory infiltrates predominantly composed by mononuclear cells in *T. cruzi*-infected mice (Figures 5(c), 5(d), 5(e), and 5(f)). The levels of PTPRC—which encodes for CD45, a pan-leukocyte marker—in heart samples were decreased in the hearts of mice treated with wild-type MSC, but not with Gal-3 knockdown MSC (Figure 5(g)). Similarly, treatment with wild-type MSC, but not with Gal-3 knockdown MSC, reduced the expression of genes in the heart which are associated with inflammation, such as IL-1*β*, IL-6, and TNF*α* (Figures 5(h), 5(i), and 5(j)). The levels of expression of IFN*γ* and T-bet, associated with Th1 responses, were significantly reduced by treatment with wild-type MSC. However, treatment with Gal-3 knockdown MSC did not reduce IFN*γ* or T-bet expression, when compared to infected controls (Figures 5(k) and 5(l)).

**Figure 6:**
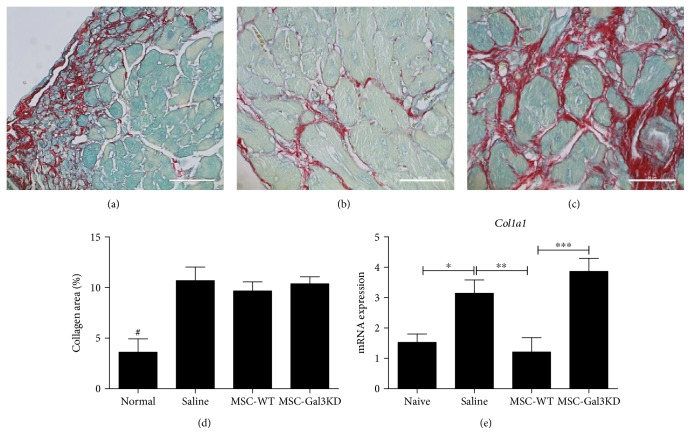
Modulation of collagen synthesis in the heart after administration of MSC. Representative images of Sirius red stained heart sections of *T. cruzi* infected mice administered with saline (a), MSC-WT (b), or MSC-Gal3KD (c). (d) Quantification of the collagen-stained area by morphometry. (e) Type I collagen (*Col1a1*) gene expression analysis by qRT-PCR in the heart tissue. ^∗^*P* < 0.05; ^∗∗^*P* < 0.01; ^∗∗∗^*P* < 0.001; ^#^*P* = 0.01, compared to the other groups.

The analysis of Sirius red-stained heart sections of *T. cruzi*-infected mice showed extensive areas of fibrosis (Figures 6(a), 6(b), and 6(c)). While the fibrosis content in the heart was not changed between the groups, collagen synthesis, as measured by collagen type I (*Col1a1*) gene expression, was reduced with wild-type MSC, but not with Gal-3 knockdown MSC (Figures 6(d) and 6(e)).

**Figure 7:**
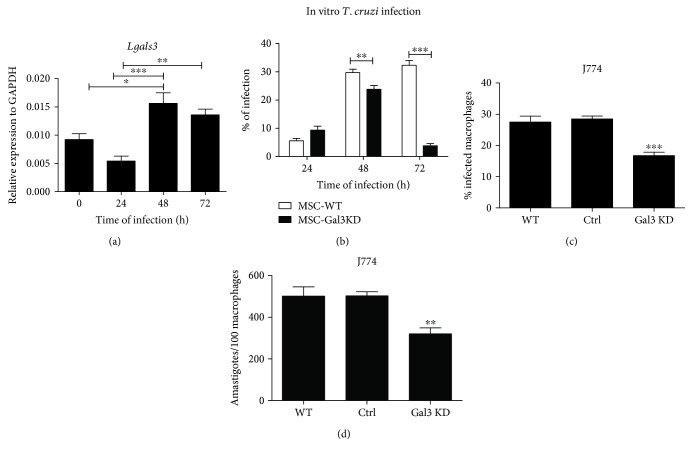
Gal-3 knockdown impairs *T. cruzi* infectivity in vitro. (a) Gene expression of Gal-3 is increased 48 h and 72 h after infection of MSC-WT with *T. cruzi*. (b) Percentage of *T. cruzi* infection in MSC-WT and MSC-Gal3KD lines during the first 72 h. (c) Percentage of infection and (d) number of parasites per cell in J774 macrophages nontransduced (WT) or transduced with control vector (Ctrl) or Gal-3 shRNA (Gal3 KD). ^∗^*P* < 0.05; ^∗∗^*P* < 0.01; ^∗∗∗^*P* < 0.001.

Since Gal-3 has been previously associated with the process of infection by *T. cruzi* [27], we hypothesized that Gal-3 is required for parasite life cycle also in MSC. In order to test that hypothesis, the MSC lines were submitted to in vitro *T. cruzi* infection. We found that Gal-3 expression is increased 48 and 72 h after *T. cruzi* infection in wild-type MSC (Figure 7(a)). Moreover, both wild-type MSC and Gal-3 knockdown MSC were successfully infected by *T. cruzi*, presenting a similar percentage of infection and number of parasites per cell 24 h after infection. However, at 48 and 72 h after infection, Gal-3 knockdown MSC presented a significantly lower percentage of infection and number of parasites per cell (Figure 7(b)). At the same time points evaluated, no differences were observed regarding the percentage of proliferating (KI67^+^) and dead cells (PI staining), between the infected MSC lines (data not shown). In order to evaluate if this was a cell-type specific effect, infection was performed also in J774 macrophages. Gal-3 knockdown was also associated with a lower percentage of infection and number of parasites per cell in J774 macrophages (Figures 7(c) and 7(d)).

**Figure 8:**
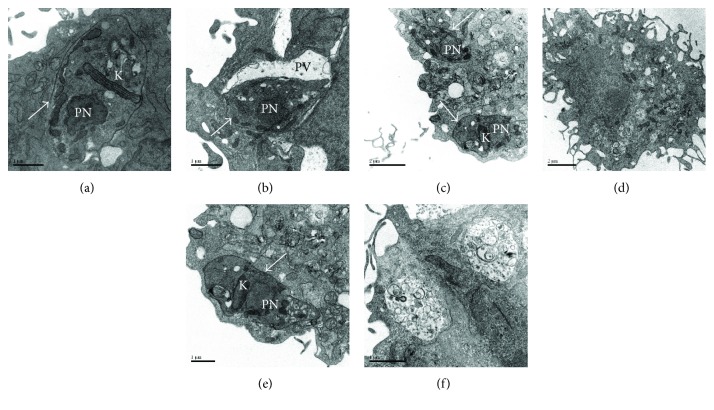
Ultrastructural analysis of *T. cruzi* infected MSC. Viable parasites were found in the cytosol of MSC-WT (a) and inside parasitophorous vacuoles of MSC-Gal3KD (b), 24 h after infection. (c and e) Viable parasites are seen in the cytosol of MSC-WT 72 h after infection. (d and f) Absence of viable parasites and presence of large vacuoles containing degraded material in the MSC-Gal3KD 72 h after infection. White arrows = viable parasites'; K = kynetoplast; PN = parasite nucleus; PV = parasitophorous vacuole.

Ultrastructure analysis by transmission electron microscopy was performed in MSC infected with *T. cruzi*, showing that *T. cruzi* efficiently evade the parasitophorous vacuoles and multiply in the cytosol in wild-type MSC (Figures 8(a), 8(c), and 8(e)). In contrast, *T. cruzi* remained inside the vacuoles in Gal-3 knockdown MSC and were frequently observed destroyed in the following days (Figures 8(b), 8(d), and 8(f)).

## 4. Discussion

Gal-3 is a multifunctional lectin with diverse, concordant, and occasionally opposing actions, when expressed by different cell types and either in extracellular or intracellular compartments [6]. Adhesion, proliferation, and migration are processes that are consistently favored by Gal-3 expression in different cell types, and increased Gal-3 expression play a role in migration and invasion by neoplastic cells [28]. In the present study, we showed that Gal-3 knockdown in MSC was associated with decreased migration and proliferative capacity. These results are in accordance with a recent study using bone marrow-derived MSC obtained from miniature pigs [29]. Here, we showed that Gal-3 plays key roles supporting cell proliferation, migration, and survival, with an impact in therapeutic effects observed after transplantation in a mouse model of chronic Chagas disease cardiomyopathy.

The process of migration and homing of MSC inflammatory sites is still poorly understood and may involve different adhesion molecules, chemokines, and receptors, such as the CXCR4/SDF-1 axis [30]. Gal-3 was recently found to promote migration of MSC through inhibition of RhoA-GTP activity, enhancement of p-AKT (ser473) expression, and regulation of p-Erk1/2 levels [29]. Based on these data and in our findings, it is reasonable to suggest that Gal-3 plays a significant role in MSC migration and homing, which could have several implications in a cell transplantation setting.

In the chronic Chagas disease model, Gal-3 expression by MSC was associated with increased migration from the peritoneal cavity to the spleen. The spleen was also characterized as a reservoir for inflammatory monocytes that emigrate from the subcapsular red pulp and populate inflammatory sites [31]. By reaching the spleen, MSC may be able to exert immunomodulatory actions, with systemic repercussions, as observed previously [32]. Besides regulating lymphocyte populations, MSC also were shown to promote expansion of regulatory populations of monocytes and granulocytes, known as myeloid-derived suppressor cells (MDSC), through HGF secretion [33]. By migrating to heart tissues of *T. cruzi* mice, MDSC were shown to suppress T lymphocytes present in the inflammatory infiltrate [34]. Indeed, i.p.-transplanted MSC showed negligible migration to the heart, but were still able to promote immunomodulation with detectable effects in the heart disease. In mice transplanted with Gal-3 knockdown MSC, however, in which a reduced cell migration to the spleens was observed, inflammation and fibrosis remained at the level of saline controls.

During the chronic phase of Chagas disease cardiomyopathy, different mechanisms are associated with the exacerbated immune response found in the heart, including parasite persistence and autoimmunity [13]. The ability of transplanted MSC to decrease cardiac inflammation in experimental *T. cruzi* infected mice was shown before in studies that applied systemic and local delivery routes for cell transplantation [14–16, 35]. Here, we demonstrated that transplanted MSC caused downregulation of inflammatory cytokines directly involved in the disease pathogenesis, such as TNF-*α* and IFN-*γ* [13]. The immunomodulatory effects observed in the heart tissue were not associated with a high recruitment and homing of MSC to the cardiac tissue, favoring the hypothesis that these cells exert a systemic modulatory action at lymphoid organs such as the spleen, where we did observe migration of MSC. This is corroborated by our finding that Gal-3 knockdown MSC had a significantly lower migration efficiency to the spleen and exerted a lower immunomodulatory action than wild-type MSC.

Regarding parasite persistence, in addition to its presence in the heart, it has been demonstrated that tissues rich in stromal cells, such as the adipose tissue, are reservoirs of *T. cruzi* [13, 36]. A role for MSC as reservoirs for *T. cruzi* in the human disease setting is possible, but has yet to be determined. Here, we show that MSC are efficiently infected by *T. cruzi*, which replicates with time of infection in vitro. Moreover, we found that Gal-3 does not interfere in the invasion process, but it is involved in the further steps of the parasite life cycle. Our data is in accordance with previous work that describes a role for Gal-3 in the step of parasite evasion from the parasitophorous vacuole to the cytosol in macrophages, a critical step for *T. cruzi* life cycle [21].

Gal-3 expression was increased by *T. cruzi* infection in the host cell in the present study, and in previous reports [27]. It has been demonstrated that Gal-3 overexpression induced by infection is important for the parasite cycle, since it can facilitate processes such as adhesion to extracellular matrix, host cell entry, and evasion from parasitophorous vacuole [20, 37]. However, Gal-3 overexpression induced by *T. cruzi* has been also associated with modulation of different aspects of the antiparasitic immune response, by inhibiting plasma cell differentiation and production of immunoglobulins [22] and promoting the release of immature thymocytes [23]. Whether increased Gal-3 expression by MSC contributes or not to the modulation of immune responses in the acute infection by *T. cruzi* is a question that needs further investigation.

## 5. Conclusion

In conclusion, Gal-3 is involved in the mechanisms of infection by *T. cruzi* and is a mediator of the immunomodulatory actions performed by MSC in a chronic Chagas disease cardiomyopathy model. Gal-3 knockdown decreased MSC survival, migration, and engraftment capabilities, leading to decreased therapeutic effects. Therefore, Gal-3 has the potential to be applied as a predictive biomarker, as part of the quality control on cell preparations to be therapeutically applied, but this merits further investigation.
